# Health Promotion at Local Level in Norway – Who, What, When, and How: A Response to Recent Commentaries

**DOI:** 10.15171/ijhpm.2019.01

**Published:** 2019-01-23

**Authors:** Susanne Hagen, Kjell Ivar Øvergård, Marit Kristine Helgesen, Elisabeth Fosse, Steffen Torp

**Affiliations:** ^1^Department of Health, Social, and Welfare Studies, University of South-Eastern Norway, Borre, Norway.; ^2^Faculty of Health and Welfare, Østfold University College, Halden, Norway.; ^3^Department of Health Promotion and Development, University of Bergen, Bergen, Norway.

First of all, we will thank Bekken,^1^ Fisher,^2^ Holt^3^ and Raphael^4^ for very good and inspiring commentaries. We feel honoured that the authors have taken time to analyse the article in depth and provided commentaries that not only are helpful to our research but also formulate important questions to move research forward on how to organize public health policies and particularly policies to increase equity in health.

Before moving to the comments, we find it necessary to describe the whole project the commented study and article is part of. The project “Addressing the social determinants of health among families with children” (SODEMIFA) ran from 2012 through 2016 and was funded by the Norwegian Research Council. In time, it coincided with the adoption of the Norwegian public health act (PHA), so the PHA became the backdrop of the project and it was in many ways a process evaluation of the implementation of the act in its early years. The project collected both qualitative and quantitative data. For the qualitative data collection, we included 10 municipalities in cases studies where personal interviews with local policy-makers and policy documents were the main data sources. The quantitative data consisted of two surveys to all Norwegian municipalities (n = 428) and one survey to all Norwegian counties (n = 19), called county municipalities. Results from the project based on both qualitative and quantitative data have been reported in a total of 13 publications (articles, book chapter, and reports). The current article is thus one of many publications from the project and part of a PhD project based on survey and register data at the municipal level.^5-7^

In general, we recognize two types of comments to the article; those who provide insight and suggestions on theoretical and methodological approaches that should be addressed to follow up our and other research in the future. The second category is the comments that more specifically critique the research questions and methods applied in the article. We will comment on the first category of comments before we move on to answer the critique from the second category.

In her comment, Bekken^1^ is concerned with the balance between universal and targeted measures and she argues that even though the Norwegian welfare state policy is based on universal measures, the social policy at the local level has a traditional perspective aimed at disadvantaged groups, in other words, targeted measures. She asks if the “Health in all Policies”-perspectives favour the health perspective more than the social conditions shaping good health and quality of life. Fisher^2^ has a similar perspective in his comment and makes the point that since the municipalities have the main responsibility for implementing the PHA, it can be characterized as a “place-based policy.” Within the context of the municipalities, the main focus will then be on services and intervention aimed at the inhabitants of the municipalities, rather than the broader determinants that will require political and social action to changes the social conditions that creates social inequalities in health.

Both Bekken and Fisher raise very important points, stating that a focus on the service provision will not necessarily reduce social inequalities. Nevertheless, Marmot^8^ makes the point that access to universal services of good quality will prevent inequalities in health. Nordic municipalities are among the most decentralised in the western world, meaning that they have the responsibility for providing the bulk of welfare services.^9^ Baldersheim et al^10^ even speak of the “local welfare state” in the Nordic countries. Municipalities have the responsibility to provide almost all individual services (eg, day care facilities and primary education), infrastructural services (eg, water and sewage) as well as planning, including land use planning and the development of local areas, which in turn include facilitating for industry and work places. Norwegian municipalities are the most decentralised even among the Nordic countries.^10^ Municipalities constitute democratic entities and on the background of local preferences, the municipal councils decide on the use of the municipal income and, for instance, they can decide to improve the quality of their childcare or to grade the payments for childcare dependent on family income. The municipal level thus balances inequalities in individual income. In recent interviews with national policy-makers, we even found that reducing social inequalities is no longer a controversial issue (unpublished manuscript). It is rather institutionalised as an element of public health with a clear focus on the broader social determinants of health. This is made possible by the communication and very close connection between the PHA and the Planning and Building Act, which also states that reducing social inequalities locally is a goal.

Holt^3^ is following up on these issues but she is framing them from a slightly different perspective. She takes her point of departure in one of the main findings of the article, namely that we found a negative correlation between the employment of public health coordinators (PHCs) in Norwegian municipalities and consideration of a fair distribution of social and economic resources between social groups in local health-promotion initiatives. In the article, this finding relates to the role of the PHCs in the municipalities. In many cases, they have part time positions, which provide them with little time to coordinate public health work between sectors. Additionally, most are employed in the health sector and not with the CEO staff or planning department,^5^ which would have provided them with more authority. Even though Holt accepts these explanations, she finds them too narrow in explaining the lack of implementation in the municipalities. The “Health in all Policies”-approach builds on an assumption that issues of social inequalities should be regarded as a health issue and that other sectors than health should adjust to this perspective and that inter-sectoral action should have this perspective as their point of departure. This is what has been termed “health imperialism.” Holt suggests that not “health” but “living conditions” might be the concept to be applied to achieve a stronger focus on how *all* sectors can contribute to reduce social inequalities by addressing the social determinants of health. We find Holt’s^3^ arguments interesting, and we believe that it should be taken into consideration when studying local political and administrative conditions for implementation of policies to reduce social inequalities at the local level.

The second category of comments is a critique of the study design on which the current commented article is based on. Raphael^4^ argues in his comment that health promotion is a complex activity that requires analytic methods recognizing the contested nature of its definition, the barriers and supports for such activities, and its embeddedness within the politics of distribution. He suggests that the complexity of health promotion activities can be best captured through qualitative methods employing open-ended questions and thematic analysis of responses.

We agree with Raphael^4^ that health promotion, and particularly policies to reduce social inequalities in health are complex and “wicked.” We further agree that qualitative studies are suitable for capturing and understanding these complexities. As shown above, the SODEMIFA project, of which the current study is a part, has in fact its main emphasis on qualitative case studies (see for example Grimm,^11^ Fosse,^12,13^ Schou,^14^ Bjørnsen,^15^ Oldeide,^16^ Shandize^17^). However, we do not share Raphael’s views on the lack of suitability of surveys and quantitative data in investigations of public health policies. On the contrary, we believe there is a need for *both* qualitative and quantitative data in order to gain knowledge of how public health policy practices are performed at a local level, as well as establishing an understanding of how policies work on multiple societal levels.

Regarding the use of quantitative data from surveys, it is a long and well-established tradition in the social sciences of using surveys to document and to produce knowledge about social phenomena. Granted, survey and questionnaire-based methods have to a lesser extent been used to assess the effectiveness of public health policy making. Despite the relative lack of survey-based studies on public health policy, we believe that ignoring this tradition and the knowledge survey- and questionnaire-based studies can produce would be a major drawback to any research effort into understanding the consequences and implications of public health policies. We assume that Raphael agrees with us on this, as he acknowledges the differences between qualitative-based and quantitative-based research enquiries as one being that of “depth vs. breadth.” Therefore, it is in the sense of ‘breadth’ that our quantitative study has provided insights into the consequences of two public health policies (employing PHCs and developing health overviews). Our study does not – as Raphael points out – give any details on how health coordinators interpret and understand the role and function of health coordinators (which is an interesting research project in its own regard). However, what our study lacks in depth it gains in breadth by assessing the associations between municipalities’ public health policies and the fair distribution in political decision-making and health promotion initiatives among social groups.

As with all survey-based studies, our study has collected information on the conditions at local level in Norway at the given time of the surveys. These data are collected without the intervention of the researchers, so the researcher bias that may occur during qualitative data collection is less of an issue in our study. By including all the municipalities, we have been able to describe how public health work is organized, and how public health policies are addressed in Norwegian municipalities both before and after the enforcement of the PHA. We have identified factors that possibly influence factors of importance for the reduction of social inequalities in health. For instance, we have found that most municipalities have a PHC, but that the effect of PHCs on public health may be questioned and needs to be investigated further (see comments above).^5,7^ For prioritizing living conditions in local health promotion, we have shown that political ideology (left wing vs. right wing) of municipal mayors seem to have no effect, whereas the presence of established cross-sectorial working groups or inter-municipal collaboration is of great importance.^6^ Municipalities that have developed health overviews have a higher prioritization of fair distribution among social groups in local policy-making compared to municipalities without health overviews.^7^ This type of knowledge is based upon quantitative data from the majority of Norwegian municipalities. Such an overview of the effects of health promotions policies would have been difficult to establish with qualitative data only.

So, to sum up our response to Raphael’s critique of our methods: we believe in a thriving research environment utilizing both qualitative and quantitative data to identify how public policy making is enacted at local levels as well as identifying the consequences and implications of different public health policies at multiple societal levels. Like a number of other researchers^18^ we strongly hold that methodological and analytical diversity is a strength that we ought to embrace also in this field of research.

## Ethical issues

Not applicable.

## Competing interests

Authors declare that they have no competing interests.

## Authors’ contributions

All the authors contributed equally to the writing of this paper.

## Authors’ affiliations

^1^Department of Health, Social, and Welfare Studies, University of South-Eastern Norway, Borre, Norway. ^2^Faculty of Health and Welfare, Østfold University College, Halden, Norway. ^3^Department of Health Promotion and Development, University of Bergen, Bergen, Norway.
